# Supplementary material for: Patterns of Intron Gain and Loss in Fungi
Source: PLoS Biol. 2004 Nov 30;2(12):e422. doi: 10.1371/journal.pbio.0020422 (PMC532390; doi:10.1371/journal.pbio.0020422)
Supplement: Table S1 — Also available at http://genes.mit.edu/NielsenEtAl/. (4.3 MB ZIP). [file pbio.0020422.st001.zip › NielsenEtAl/html/176.html]

AN1006.1.NCU05298.1.MG06062.1.FG01947.1


```
 CLUSTAL W (1.82) Multiple Sequence Alignments - Introns Inserted


Sequence 1: MG06062.1	911 aa
Sequence 2: FG01947.1	884 aa
Sequence 3: NCU05298.1	984 aa
Sequence 4: AN1006.1	873 aa
Alignment Length: 990 aa
Number Identitical Residues: 376 aa
Alignment Score (without introns) 20290


MG06062.1 	----------------------------------------MGTIVGLPP-----IPTEAP
NCU05298.1	MEAPALEQRQSLHDSSERQQRFTSLILPNGAAAAAAEKNPRGAADYCPSDNDNDIDNDLA
FG01947.1 	------------------------------------------------------------
AN1006.1  	------------------------------------------------------------
          	                                                            

MG06062.1 	PSYTTVKK~LDIFPPSPPDTAKESRQHSRRSSISVGTGLVEDVDCAVVNDEASRP-YPLP
NCU05298.1	STRTASPT~TTDFSSSSSDDNSTTLETSVNYSHSSNTNTNTSCPPSPITSSSLKPAYPLP
FG01947.1 	--MSTQRT0AHSLPPSPPSTAKPSRIGSFHDLQDIPS-----VDQSCL--DSIKP-YPLP
AN1006.1  	---MSTTV~TQVRTGSIPKTLKTSQIR---------------VEEQEITELDTAD-IPLP
          	    :        . * ..  . :                       :..       ***

MG06062.1 	PP-TRQNNVLPQDLKTPDSHVERDPRLIRLTGIHPFNTEAPLSDLFDEG~FINSKDLHYV
NCU05298.1	PPSTRLTTILPTDLKTPDHLIPRDPRLIRLTGSHPFNVEPPLTALFEHG~FLTPQNLHYV
FG01947.1 	PR-CSSKSVLKEDLKTPDSFVERDPRLIRLTGVHPFNVEAPLSDLYDEG~FLTSENLHYV
AN1006.1  	PPSKEPTEVLSLDKTTPDSHVPRDPRLIRLTGVHPFNVEPPLTALFQQG1FLTPPELFYV
          	* :   . :*  * .***  : ********** ****.*.**: *::.* *:.. :*.**

MG06062.1 	RNHGAVP-----------RVEDADMMDWEFTVEG~MVESPMKLTLRTLMK--DFAQATYP
NCU05298.1	RNHGPIPSSVATPPATINKEEDDSLLNWEFTVEG~LVEHPLKISVRELMDASKWDNVTYP
FG01947.1 	RNHGHVP-----------RCEDDEILDWEFEIDG~LVETPIKMTVRDLIN--EYQQLTYP
AN1006.1  	RNHGPVP-----------HVRDEDIPNWELRIEG2LVEKPITLSFKQILQ--NYDQITAP
          	**** :*           : .* .: :**: ::* :** *:.::.: ::.  .: : * *

MG06062.1 	VTLVCAGNRRKEQNVVRKTKGFSWGAAGLSTALWTGVPLGDLLARAMPKR----GARYVY
NCU05298.1	VTLVCAGNRRKEQNVLRKSKGFSWGAGGLSTALWTGVGLSEILARAKPLTKKGGGARYVC
FG01947.1 	VTFVCAGNRRKEQNIVRKTKGFSWGAAGLSTALWTGVAIGDLLAAAKPKR----GARYVC
AN1006.1  	ITLVCAGNRRKEQNTVRKSKGFSWGSAALSTALFTGPMMADIIKSAKPLR----RAKYVC
          	:*:*********** :**:******:..*****:**  :.:::  * *       *:** 

MG06062.1 	FEGADKL~PNGYYGTSIKLNWVVDPNRGIMVAHRMNGEALHPDHGKPLRIVIPGQIGGRS
NCU05298.1	FEGADQL~PNGTYGTSVKLAWAMDPNKGIMVAHKMNGENLHPDHGRPVRVVVPGQIGGRS
FG01947.1 	FEGADKL~PNGYYGTSIKLNWCMDPNRGVMVAHRMNGKTLHPDHGKPVRLVIPGQIGGRS
AN1006.1  	MEGADNL0PNGNYGTSIKLNWAMDPNRGIMLAHKMNGEDLRPDHGRPLRAVVPGQIGGRS
          	:****:* *** ****:** * :***:*:*:**:***: *:****:*:* *:********

MG06062.1 	VKWLKRIVVTSEPSDNWYHIYDNRVLP~TMVTPEAS----ADLPDTWKDERYAIYDLNTN
NCU05298.1	VKWLKRIVVTKGPSENWYHVFDNRVLP~TTVGPEESGEKTEEMERVWRDERYAIYDLNVN
FG01947.1 	VKWLKKITVTQEPSDNWYHIYDNRVLP~TMVSPEES----ADLPEVWKDEKYAIYDLSTN
AN1006.1  	VKWLKKLIITDAPSDNWYHIYDNRVLP2TMVTPDMS----SQNPSWWRDERYAIYDLNVN
          	*****:: :*. **:****::****** * * *: *     :    *:**:******..*

MG06062.1 	SATCYPAHDEKVPITA--GAGVYKFRGYAYAGGGKRVTRMEVTLDKGKS~WRLAKMIYPE
NCU05298.1	SVICEPGHGEVVSLRGDEGAGTYRLRGYAYAGGGRRVTRLEVTLDQGKS~WRLAGIEYPE
FG01947.1 	SAICYPAHQESVTLTN--TSESYKVRGYAYGGGGRRITRVEITLDKGKS~WRLANIRYHE
AN1006.1  	SAAVYPQHKETLDLAA--ARPFYTAKGYAYAGGGRRITRVEISLDKGKS1WRLARIEYAE
          	*.   * * * : :        *  :****.***:*:**:*::**:*** **** : * *

MG06062.1 	DDYRLASVDDTLYGGKVDLSWREASFCWCFWELDIPTTDLVAADDVMIRAMDDSMMVQPR
NCU05298.1	DRYREAQDGEELFGGRLDVSWRESCFCWCFWDLEIPLSELRKAKDVCIRAMDESLALQPK
FG01947.1 	DDYRNAPEGDTLYGGRVDMWWRETSFCWCLWDLEIPLDELRNADDIMMRAMDESMNIQPR
AN1006.1  	DKYRDFEG--TLYGGRVDMAWREACFCWSFWSLDIPVSELASSDALLVRAMDEALSLQPK
          	* **       *:**::*: ***:.***.:*.*:**  :*  :. : :****::: :**:

MG06062.1 	DMYWSVLGMMNNPWYRVMIHKEGHDLRFEHPTQPALIPGGWMERVKKAGGDLSNGFWGER
NCU05298.1	EMYWSVLGMMNNPWFRVVIHHEGDTLRFEHPTQPMLTSDGWMDRVKKEGGNLANGFWGEK
FG01947.1 	DMYWSVLGMMNNPWFRIVIHKDDNALRFEHPTHPTLKNKGWMERVKEDGGNLTNGYWGEK
AN1006.1  	DMYWSVLGMMNNPWFRVKITNENGRLLFEHPTDITG-SSGWMEQIKKAGGDLTNGNWGER
          	:*************:*: * ::.  * *****.      ***:::*: **:*:** ***:

MG06062.1 	VAGDEEESAAVAEEPANEICMTNPSVNKTITLDELKAHDSESQPWFVVDGQVYDGIKFLE
NCU05298.1	VPG--AEENVVKEEPVKEISMVDEKVTRLITLEELRQHDGEEEPWFVVNGQVYNGTPFLE
FG01947.1 	APG--EVKEVVVKELEKQICLTNPHINREITIEELKAHEGEDEPWFVVNGEVYDGTPYLS
AN1006.1  	QEG----EEPVEAEPVVEVNMKKEGVTRIIDLEEFKKNSSDERPWFVVNGEVYDGTAFLE
          	  *    .  *  *   :: : .  :.: * ::*:: :..:..*****:*:**:*  :*.

MG06062.1 	GHPGGAASIIGAAGQDCSEEFLTIH1SENAKAMMPEYHIGSLDEKSRSLLAEPESTQPES
NCU05298.1	GHPGGAASITGAAGQDVTDEFLAIH1SENAKAMMPTYHIGTLTPSALAALKSSSTSDPAL
FG01947.1 	GHPGGAASIFGAAGQDATEEFMAIH1SENAKAMLPTYHIGTLNKESRAILSG-DATATSD
AN1006.1  	GHPGGAQSIISAAGTDASEEFLEIH1SETAKKMMPDYHIGTLDKASLEALRKGNADTTDS
          	****** ** .*** * ::**: ** **.** *:* ****:*   :   *   .:  .  

MG06062.1 	TEP-RPVFLQSRTWSKALLQEKISVSGDSKIFSFKLDHAEQAIGLPVGQHLMMRLRDPAT
NCU05298.1	SDPSRPIFLQSKTWNSAILTFKESVSPDTKIFHFALSHPAQSIGLPVGQHLMMRLPDPAK
FG01947.1 	DSN-REIFLQSKTWSKALLCKKMSISPDTKIFSFKLNHEGQKIGLPTGQHLMMRLRDPAT
AN1006.1  	SSDPRPTFLTPKAWTKATLTKKTSVSSDTHIFTLSLEHPSQALGLPTGQHLMLKTPDPKS
          	 . .*  ** .::*..* *  * *:* *::** : *.*  * :***.*****::  ** .

MG06062.1 	--REAIIRAYTPLSDCVVEKGLLRVLVKIYYKSA--GMEGGKMTQALDHLPVGHWVDFKG
NCU05298.1	P-TESIIRAYTPISDGTLERGTLRVLVKIYYASPTEDIKGGQMTQALDALALGKAVEFKG
FG01947.1 	--REAIIRSYTPYSDGS-DCGQLDILIKIYYDTP--QRKGGAMTQALDSLPVGHWVDFKG
AN1006.1  	SSSGSIIRSYTPISPSD-QLGMVDILIKIYAETP--SIPGGKMTTALDTLPLGSVIECKG
          	.:  :***:*** *    : * : :*:***  :.     ** ** *** *.:*  :: **

MG06062.1 	PVGRFEYLGKGKCRVSGKERSVRRFIMICGGSGVTPIFQVFRAVMQDKEDQTRCLVLDGN
NCU05298.1	PVGKFVYQGRGVCSVNGRERKVKRFVMVCGGSGVTPIYQVLRAVAVDDQDGTECLVLDGN
FG01947.1 	PVGKFVYHGNGRCIISDRERRVRRFIMVCGGSGITPIRQVLRAVMQDPSDTTPCIVFNGN
AN1006.1  	PTGRFEYLDRGRVLISGKERFVKSFVMICGGTGITPVFQVLRAVMQDEQDETKCVMLDGN
          	*.*:* * ..*   :..:** *: *:*:***:*:**: **:***  * .* * *::::**

MG06062.1 	RTEEDILCKDELDKLAVGN--EDRCRLVYSLSKPSESWTGAKGRMDRALFEKELGVSGSN
NCU05298.1	RVEGDILMKSELDELVERAKPEGRCRVKYTLSRPGAEWEGLRGRLDKTMLEREVGEGDLR
FG01947.1 	RSFQDILCKEELDNLEAAN--PSKCRIINALTDPPPDWSGIKGFVNQALVPEQMALPKPS
AN1006.1  	RLEEDILLKNELDEFEALAGKKEKCKIVHTLTKGSESWTGRRGRIDEELIRQHAGTPD--
          	*   *** *.***::        :*::  :*:    .* * :* ::. :. .. .     

MG06062.1 	SQ--DMVLICGPESMEKSVKQILTQELGWKDDDLLFF
NCU05298.1	GET--MVLLCGPEGMQNMVREVLKG-MGWKDEDVLVF
FG01947.1 	GEGDELVLVCGPPPMVKAAEATFLG-LGFKSDDMVFF
AN1006.1  	RET--MVLVCGPEAMEKASKKILLS-LGWKEENLHYF
          	 :   :**:***  * :  .  :   :*:*.:::  *
```
